# A Rare Case of Clear Cell Sarcoma of the Tongue: A Case Report

**DOI:** 10.7759/cureus.50739

**Published:** 2023-12-18

**Authors:** Noura Seghrouchni, Achraf Miry, Zainab El Zouiti, Nassira Karich, Adil Abdenbitsen, Amal Bennani

**Affiliations:** 1 Laboratory of Pathology, Faculty of Medicine and Pharmacy of Oujda, Mohammed First University of Oujda, Oujda, MAR; 2 Department of Maxillofacial Surgery, Faculty of Medicine and Pharmacy of Oujda, Mohammed First University of Oujda, Oujda, MAR

**Keywords:** oral cavity neoplasms, sarcoma, soft part melanoma, oral tongue, clear cell sarcoma

## Abstract

Clear cell sarcoma (CCS), previously known as soft tissue melanoma due to similarities with melanoma, is a rare and aggressive neoplasm. This tumor predominantly occurs in the lower limbs and rarely affects the tongue, as well as other head and neck locations. To our knowledge, only five cases have been reported in the English literature. CCS presents many similar morphological and immunohistochemical features to those of melanomas, sarcomatoid cell carcinoma, angiomatoid histiocytoma, and Ewing sarcoma, which makes the diagnosis difficult, especially in cases of uncommon locations. The treatment is based on oncological surgery and adjuvant radiation therapy as these tumors show low sensitivity to chemotherapy. This study aimed to report a case of an 88-year-old male patient who presented a large, rapidly growing nodular lesion on the right border of the mobile tongue diagnosed with CCS of the tongue.

## Introduction

Clear cell sarcoma (CCS), previously known as soft tissue melanoma, is a rare and aggressive tumor that preferentially occurs in the lower extremities of young adults [1]. CCS arising in the tongue is extremely rare [2]. To our knowledge, only five cases of tongue CCSs have been reported in English literature. The term soft part melanoma is because CCS shares many similarities such as the expression of melanocytic markers. The most important distinguishing features between the two entities are the presence of V-Raf murine sarcoma viral oncogene homolog B1 (BRAF) mutations in melanomas and their absence in CCS. In addition, CCS is characterized by the presence of rearrangement of Ewing sarcoma breakpoint region 1 (EWSR1), whereas it is absent in melanomas [3,4].

As an uncommon entity, its diagnosis can be difficult, relying on histological and molecular findings. CCS exhibits a characteristic nested or fascicular architecture with epithelioid to plump spindled cells partitioned by thin fibrous septa. It typically shows a notably infiltrative growth pattern through dense fibrous tissue.

## Case presentation

An 88-year-old male patient presented with a large, rapidly growing nodular lesion on the right border of the mobile tongue within three months. The patient had a history of diabetes mellitus under oral antidiabetics, arterial hypertension under therapy, and no history of alcohol and tobacco abuse.

**Figure 1:**
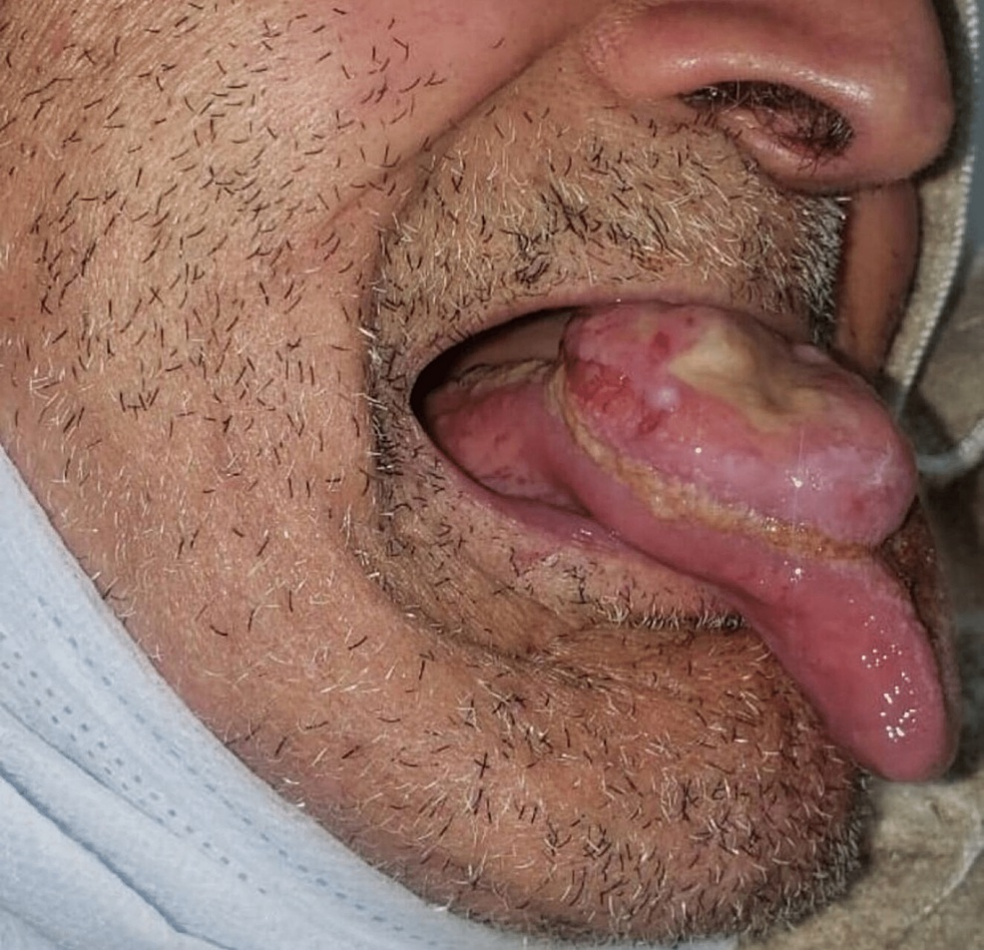
The tongue nodular lesion: a large, 4 cm x 3 cm x 1 cm focally ulcerated lesion, with the presence of many fibrinous deposits on the surface.

**Figure 2:**
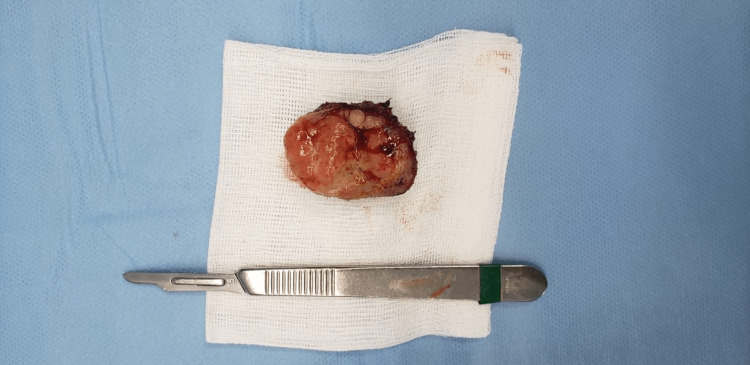
The resection specimen.

Oral cavity examination revealed the presence of a large, focally ulcerated nodular lesion, measuring 4 cm x 3 cm x 1 cm with a firm consistency, and the presence of many fibrinous deposits on the surface of the lesion (Figure 1). No cervical lymphadenopathies were clinically found. The patient underwent a large resection of the lesion (Figure 2).

**Figure 3:**
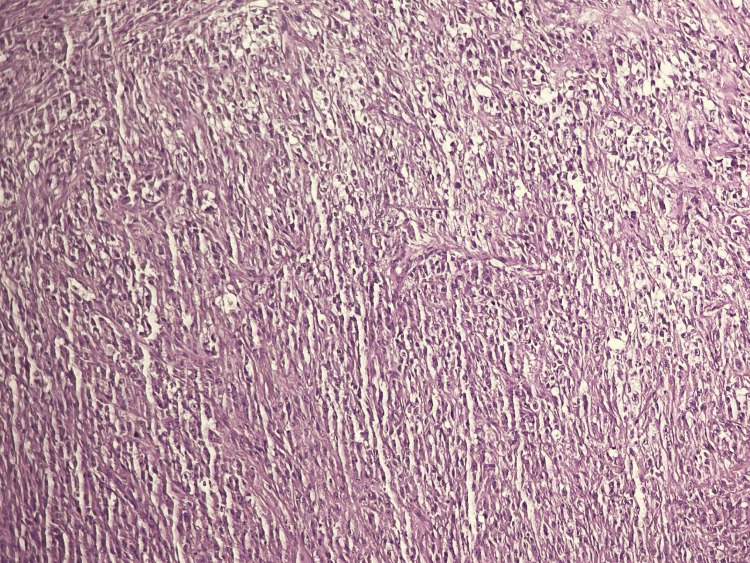
Microphotography showing nests of epithelioid spindle cells with clear eosinophilic cytoplasm (H&E, x200). H&E, hematoxylin and eosin stain

**Figure 4:**
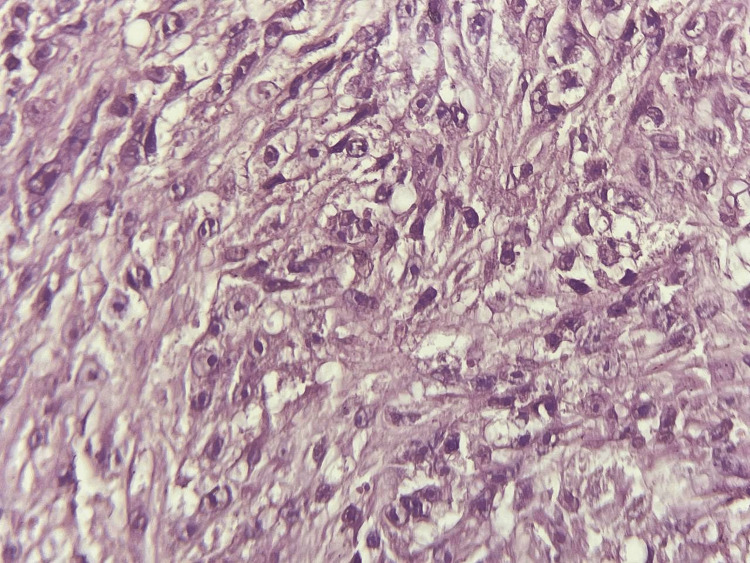
Microphotography showing tumor cells with prominent nucleoli and a clear eosinophilic cytoplasm (H&E, x400). H&E, hematoxylin and eosin stain

**Figure 5:**
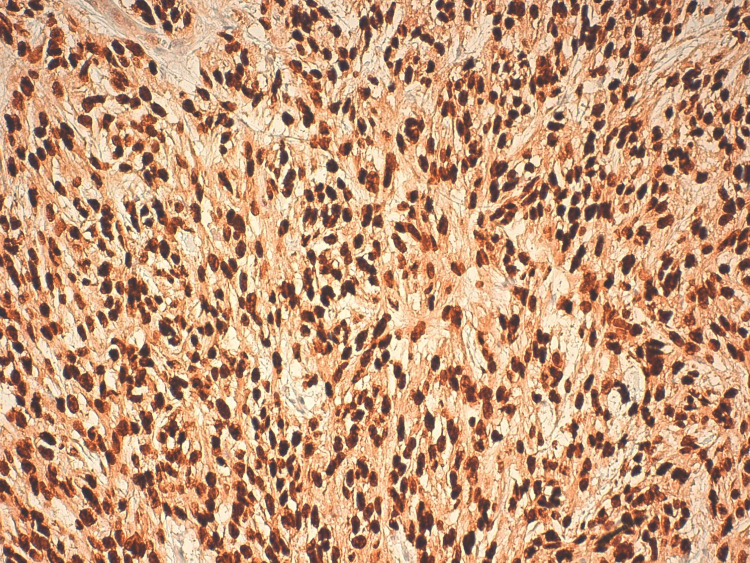
Neoplastic cells showing nuclear expression of SOX10.

The pathological assessment revealed a nested proliferation of large, epithelioid, clear, and eosinophilic cells showing prominent nucleoli and vesicular chromatin. Nests of tumor cells were separated by fibrous septa (Figures 3-4). Many foci of perineural invasions were observed. Only three mitoses per 10 high-power fields could be identified. The resection margins were free of proliferation. The immunohistochemical study showed expression of S100, HMB-45, SOX10, and vimentin and no expression of melanoma antigen-A (Melan-A), epithelial membrane antigen (EMA), cytokeratin, p63, CD86, or CD45 (Figure 5).

Fluorescent in situ hybridization revealed the presence of EWSR1 and ATF1 gene fusion.

The patient underwent adjuvant radiotherapy. Further investigations revealed no distant locations and no metastatic lymphadenopathies. The six-month follow-up indicated favorable progress, with no reported recurrences or metastases in the patient.

## Discussion

CCS is a rare sarcoma of soft tissue. The name *melanoma of soft parts* was given to this entity following the identification of melanocytic differentiation. The melanocytic differentiation has been identified at the ultrastructural level by the presence of melanosomes and at the immunohistochemical level by the identification of expression of melanocytic markers such as HMB-45, S100, microphthalmia-associated transcription factor (MITF), tyrosinase, and Melan-A [5].

More classical locations for CCS include deep soft tissue of the distal extremities, especially the lower limbs. Additionally, it may occur in the proximal extremities, trunk, and, rarely, the skin [11]. This latter location is well documented, although some cases of cutaneous CCS can result from the extension of deep-seated lesions [12].

The occurrence of CCS is extremely rare in the head and neck region, and it occurs even rarely in the tongue, with, to our knowledge, only five reported primary cases of CCS in the tongue documented in the English literature (Table 1).

**Table 1:** Clinical, morphological, immunohistochemical, and molecular features of clear cell sarcoma cases in the English literature. EWSR1, Ewing sarcoma breakpoint region 1 [6,7,8,9,10]; ATF1, activating transcription factor 1 [6,7,8,9,10]; FISH, fluorescent in situ hybridization [6,7,8,9,10]; F, female [6,7,9,10]; M, male [8]; +, positive; -, negative; *Foc, focally [9]; Melan-A, melanoma antigen-A

Reference	Age (years)	Sex	Clinical features	Morphology	Immunohistochemistry	Genes implicated in fusion (Technique)	Treatment	Follow-up
Kraft et al., 2013 [6]	82	F	Tongue lesion (no further details) and cervical adenopathies	Epithelioid and spindle cells Positive lymph nodes	S100+, other melanocytic markers -	EWSR1-ATF1 (FISH)	Total glossectomy, lymph nodes dissections, and radiation therapy	Good evolution No recurrence
Singh et al., 2013 [7]	29	F	Nodular lesion; tip of the tongue	Spindle cells; perineural invasions	S100+, cytokeratin –, Melan-A -	EWSR1-ATF1 (FISH)	Surgery	Good evolution No recurrence
Feasel et al., 2016 [8]	44	M	Nodular lesion of the tongue base	Spindle and epithelioid; focal pseudo-alveolar pattern	S100+, CD68+, HMB-45+, Melan-A -	EWSR 1 (FISH break-apart probe)	Surgery and radiation therapy	Good evolution
Breton et al., 2018 [9]	44	F	Mobile tongue mass	Epithelioid clear cytoplasm	S100+, SOX10+, CD56 Foc+*, HMB-45-, Melan-A-	EWSR1-ATF1 (FISH)	Surgery and radiation therapy	Not provided
Baus et al., 2018 [10]	44	F	Mass of the ventral tongue	Clear spindle cells	S100+, synaptophysin+, CD56+ HMB-45-, Melan-A-	EWSR1-ATF1 (FISH)	Surgery and radiation therapy	Good evolution No recurrence
This study	88	M	Nodular mass of the ventral tongue 4 cm greater dimension	Epithelioid cells	S100+, HMB-45+, SOX10+, vimentin+, Melan A-	EWSR1-ATF1 (FISH)	Surgery and radiation therapy	Good evolution

CCS of the tongue, similar to most reported cases of mucosal CCS of the head and neck region, tends to occur in young patients. It exhibits a distribution regarding sex and age similar to that observed in deep soft tissue cases [8].

Clinically, the tumor most commonly presents as a slowly growing mass in the deep soft tissue. Mucosal CCS can sometimes be mistaken for a benign lesion such as a case of gingival CCS mistaken for an odontogenic abscess in a 17-year-old girl [8]. For mucosal CCS, such as CCS of the tongue, since it tends to be superficial, the diagnosis can be made relatively early in the stage of small lesions. In our reported case, the lesion was large as it was neglected by the patient.

Morphologically, a nested proliferation with numerous fibrous septa is often observed. The cells are large and epithelioid, featuring clear to eosinophilic cytoplasm with prominent nucleoli and vesicular chromatin [8].

At the immunophenotypic level, CCS cells show a diffuse expression of S100 protein and SOX10 and frequently show expression of melanocytic markers (HMB-45, Melan-A, Mel-CAM, and MiTF) [8].

Molecular biology is often necessary for a positive diagnosis, especially to distinguish it from melanoma [13]. Fluorescent in situ hybridization (FISH) enables the identification of the EWSR1 gene fusion with the ATF1 gene, secondary to the translocation t(12;22) (q13; q12). FISH enables confirmation of diagnosis in 70% of CCS cases. Molecular biology study also includes the identification of EWSR1-ATF1 fusion transcripts by reverse transcription polymerase chain reaction (RT-PCR). It is positive in more than 90% of CCS cases [14,15].

Many entities are included in the differential diagnosis. The most important and challenging diagnosis remains melanoma, particularly its nodular variant. This is noteworthy because nodular melanoma appears to be overrepresented in the head and neck region, with an incidence of 20%, compared to 12% in other sites [7,15]. In addition, the diagnosis can be morphologically more challenging in cases of nodular melanoma lacking a radial growth phase, therefore showing only a nested pattern as that present in CCS cases. Similarly, nodular melanoma cases with clear cell change add more difficulties to the diagnosis [16]. It is worth mentioning that even though rare, CCSs with a junctional component exist and may be easily confused with the diagnosis of melanoma [3]. Immunohistochemical features cannot help distinguish these two entities since they both express the same melanocytic markers. In addition to melanoma, other melanocytic lesions could be confused with CCS such as cellular blue nevus and perivascular epithelioid cell neoplasm (PEComa).

The gastrointestinal neuroectodermal tumor (GNET) is another important entity to include in the differential diagnosis. It is a rare tumor that occurs in the small intestine, stomach, or colon wall with morphological overlap with features of CCS. However, this neoplasm does not express melanocytic markers [17,18].

Despite the many overlapping features between the two entities, the pathologist should pay attention to some distinguishing features such as the presence of osteoclast-like giant cells and the more pleomorphic aspect of the nuclei, two elements that are suggestive of the diagnosis of GNET since cases of CCS show more monotonous cells and classically no osteoclast-like giant cells. Immunohistochemistry plays a key role in distinguishing between CCS and GNET since GNET classically lacks expression of melanocytic markers. On the molecular level, GNET shows rearrangement of the EWSR1 gene; however, fusion does occur with the CREB1 gene and not with the AATF1 gene (as in the case of CCS) [8]. However, EWR1-CREB1 fusions have been documented in 6.3% of CCS.

While the rearrangement of EWSR1 helps obtain a definitive diagnosis in most cases of CCS, it is not specific to this entity and is reported in an increasing number of soft tissue tumors that can present in the head and neck region. These include angiomatoid fibrous histiocytoma, Ewing sarcoma, and myoepithelioma of soft tissue [19].

Other differential diagnoses include sarcomatoid squamous cell carcinoma, which is highly aggressive. The diagnosis can be oriented by expression of high-molecular-weight cytokeratin, p63, and p14. Other sarcomas that may be confused with CCS are cited in Table 2, with the respective immunohistochemical stain that should be used to retain or exclude them [8].

**Table 2:** Principal differential diagnoses with useful immunohistochemical stains. +, positive; -, negative

Entity	Cytokeratin	Melan-A	CD68	CD99	Desmin	P63
Clear cell sarcoma	-	+	+	+	-	-
Melanoma	-	+	+	+	-	-
Sarcomatoid cell carcinoma	-	-	-	-	+	-
Angiomatoid histiocytoma	-	-	+	-	-	-
Ewing sarcoma	-	-	-	+	-	-

The main treatment of CCS in its classical location, as well as for those located on the tongue, remains based on surgical resection. The overall five-year survival rate is approximately 50%-60%, with factors such as tumor necrosis and a size greater than 50 mm being indicators of bad prognosis. A long-term follow-up is necessary as cases of recurrence and distant metastases have been reported [19].

## Conclusions

CCS is a rare and aggressive soft tissue tumor. It occurs preferentially in deep soft parts of the lower limbs in young adults. Primary tongue CCS is extremely rare. Many entities are included in the differential diagnosis of CCS, including the challenging melanoma, especially in its nodular form, as both entities express melanocytic markers and show the presence of melanosomes at the ultrastructural level. Molecular biology is often necessary to establish a definitive diagnosis, especially in uncommon locations such as the tongue, which enables identification of t(12;22) (q13; q12) with secondary EWSR1-ATF1 fusion. The treatment is based on surgery followed by a long-term follow-up, as recurrences and distant metastases have been reported in cases of treated CCS.

## References

[1] Weiss SW, Goldblum JR, Folpe AL (2008). Malignant Tumors of the Peripheral Nerves. In: Enzinger and Weiss’s Soft Tissue Tumors.

[2] Kosemehmetoglu K, Folpe AL (2010). Clear cell sarcoma of tendons and aponeuroses, and osteoclast-rich tumour of the gastrointestinal tract with features resembling clear cell sarcoma of soft parts: a review and update. J Clin Pathol.

[3] Panagopoulos I, Mertens F, Isaksson M, Mandahl N (2005). Absence of mutations of the BRAF gene in malignant melanoma of soft parts (clear cell sarcoma of tendons and aponeuroses). Cancer Genet Cytogenet.

[4] Langezaal SM, Graadt van Roggen JF, Cleton-Jansen AM, Baelde JJ, Hogendoorn PC (2001). Malignant melanoma is genetically distinct from clear cell sarcoma of tendons and aponeurosis (malignant melanoma of soft parts). Br J Cancer.

[5] Hantschke M, Mentzel T, Rütten A, Palmedo G, Calonje E, Lazar AJ, Kutzner H (2010). Cutaneous clear cell sarcoma: a clinicopathologic, immunohistochemical, and molecular analysis of 12 cases emphasizing its distinction from dermal melanoma. Am J Surg Pathol.

[6] Kraft S, Antonescu CR, Rosenberg AE, Deschler DG, Nielsen GP (2013). Primary clear cell sarcoma of the tongue. Arch Pathol Lab Med.

[7] Singh M, Ieremia E, Debiec-Rychter M, Connolly G, Calonje JE (2014). Clear cell sarcoma of the tongue. Histopathology.

[8] Feasel PC, Cheah AL, Fritchie K, Winn B, Piliang M, Billings SD (2016). Primary clear cell sarcoma of the head and neck: a case series with review of the literature. J Cutan Pathol.

[9] Breton S, Dubois M, Geay JF, Gillebert Q, Tordjman M, Guinebretière JM, Denoux Y (2019). Clear cell sarcoma or gastrointestinal neuroectodermal tumor (GNET) of the tongue? Case report and review of the literature of an extremely rare tumor localization. Ann Pathol.

[10] Baus A, Culie D, Duong LT, Ben Lakhdar A, Schaff JB, Janot F, Kolb F (2019). Primary clear cell sarcoma of the tongue and surgical reconstruction: about a rare case report. Ann Chir Plast Esthet.

[11] Enzinger FM (1965). Clear-cell sarcoma of tendons and aponeuroses. An analysis of 21 cases. Cancer.

[12] Falconieri G, Bacchi CE, Luzar B (2012). Cutaneous clear cell sarcoma: report of three cases of a potentially underestimated mimicker of spindle cell melanoma. Am J Dermatopathol.

[13] Cheah AL, Billings SD (2012). The role of molecular testing in the diagnosis of cutaneous soft tissue tumors. Semin Cutan Med Surg.

[14] Wang WL, Mayordomo E, Zhang W (2009). Detection and characterization of EWSR1/ATF1 and EWSR1/CREB1 chimeric transcripts in clear cell sarcoma (melanoma of soft parts). Mod Pathol.

[15] Patel RM, Downs-Kelly E, Weiss SW (2005). Dual-color, break-apart fluorescence in situ hybridization for EWS gene rearrangement distinguishes clear cell sarcoma of soft tissue from malignant melanoma. Mod Pathol.

[16] Dabouz F, Barbe C, Lesage C (2015). Clinical and histological features of head and neck melanoma: a population-based study in France. Br J Dermatol.

[17] Stockman DL, Miettinen M, Suster S (2012). Malignant gastrointestinal neuroectodermal tumor: clinicopathologic, immunohistochemical, ultrastructural, and molecular analysis of 16 cases with a reappraisal of clear cell sarcoma-like tumors of the gastrointestinal tract. Am J Surg Pathol.

[18] Thway K, Fisher C (2012). Tumors with EWSR1-CREB1 and EWSR1-ATF1 fusions: the current status. Am J Surg Pathol.

[19] Sara AS, Evans HL, Benjamin RS (1990). Malignant melanoma of soft parts (clear cell sarcoma): a study of 17 cases, with emphasis on prognostic factors. Cancer.

